# Emergency Departments Leading the Transformation of Alzheimer’s and Dementia Care: Emergency Care Redesign

**DOI:** 10.1111/jgs.70394

**Published:** 2026-03-25

**Authors:** Afshana Hoque, Allison Cuthel, Corita R. Grudzen, Manish N. Shah, Abraham A. Brody, Jori E. Fleisher, Jennifer DiMascio-Donohue, Katelyn McLain, Lin T. Tun, Julia Levine, Keith S. Goldfeld, Joshua Chodosh, ED-LEAD Investigators

**Affiliations:** 1 Division of Supportive and Acute Care Services, Memorial Sloan Kettering Cancer Center, New York, New York, USA; 2 Department of Emergency Medicine, University of Wisconsin-Madison, Madison, Wisconsin, USA; 3 NYU Rory Meyers College of Nursing, Hartford Institute for Geriatric Nursing, New York, New York, USA; 4 Division of Geriatric Medicine and Palliative Care, New York University Grossman School of Medicine, New York, New York, USA; 5 Department of Neurological Sciences, Rush University Medical Center, Chicago, Illinois, USA; 6 Department of Population Health, New York University Grossman School of Medicine, New York, New York, USA; 7 VA New York Harbor Healthcare System, New York, New York, USA

**Keywords:** champions, clinical decision support, dementia, emergency department, huddles, revisits

## Abstract

Over 50% of persons living with dementia (PLWD) and their care partners (dyads) visit the emergency department (ED) every year. In the ED, healthcare professionals face complex challenges managing acute issues and symptoms of Alzheimer's disease and Alzheimer's disease–related dementias without provider training or in-ED structures to ensure a successful discharge. While many of these visits are for conditions more suitable for ambulatory care, as many as 50% of PLWD discharged from the ED return within 30 days, suggesting opportunities to improve ED care, and discharge processes. Emergency Care Redesign (ECR) includes intentional workflows where physicians, nurses, and social workers engage in a team-based approach with structured assessments to manage a myriad of potential psychosocial and behavioral issues contributing to the need for ED care. Three core components comprise this evidence-based, efficient pragmatic intervention for PLWD and their care partners: (1) problem identification, (2) problem prioritization, and (3) provision of non-pharmacologic solutions supported by community resources. Although these components are essential to provide optimal ED care and reduce revisits and other adverse outcomes, they require an embedded clinical decision support structure, focused training, and clear workflows. In this paper, we describe the ECR intervention as one of three being implemented in the cluster-randomized multifactorial pragmatic trial, Emergency Departments LEading Transformation of Alzheimer's and Dementia Care (ED-LEAD), designed to improve care for PLWD and their outcomes after discharge home within 15 health systems and 79 EDs across the United States.

## 1 | Background and Significance

More than 50% of persons living with dementia (PLWD) and their care partners visit the emergency department (ED) every year [1]. Once in the ED, PLWD, their care partners, and healthcare professionals face unique and complex challenges managing acute issues and symptoms of Alzheimer's disease and Alzheimer's disease–related dementias (AD/ADRD) [2]. ED visits are complicated by difficulties identifying PLWD; unreliable histories due to memory impairment; a high prevalence of behavioral, psychiatric, and comorbid conditions [3]; polypharmacy and related adverse drug events [4]; considerable time pressures within a disorienting environment; and age-related changes in pharmacokinetics and pharmacodynamics, all of which call for geriatric, interdisciplinary care [5]. Despite motivation to deliver high-quality geriatric care, most ED providers lack geriatric expertise, and in particular, training and confidence in identifying and managing AD/ADRD [6]. Even with the development of recently created resources (see e.g., https://gedcollaborative.com/), this gap is exacerbated by the weight of communication issues that often complicate or drive an ED visit [7]. Such challenges may contribute to missed or incorrect diagnoses, ineffective or inappropriate discharge plans, and recurring ED visits with higher morbidity and mortality [8].

The ED infrastructure is well-suited to rapid triage and management of problems in which an interdisciplinary team—physicians, advance practice providers, nurses, and social workers—is often co-located. Although outpatient team-based dementia care has worked well for PLWD, team-based ED care has not historically served PLWD in an optimal fashion. Despite the many challenges, simple interventions have demonstrated promise. Brief online training modules have enhanced healthcare professionals' recognition of PLWD needs and shortened hospital stays [9, 10]. In ED settings, clinical decision support (CDS) in the form of simple pop-up alerts within the electronic health record (EHR) has improved timely, evidence-based care for conditions ranging from sepsis to opioid overdose [11, 12]. Separately, workflows using team huddles to communicate complex information in time-pressured, multidisciplinary settings—from operating rooms to the ED—have improved patient outcomes, and reduced adverse events [13, 14]. Combining elements of these approaches, an ED care intervention for individuals living with serious illness successfully leveraged the unique environment and multidisciplinary support of the ED via new and intentional workflows reinforced by digital alerts [15]. This multimodal, pragmatic approach shaped Emergency Care Redesign (ECR), one of 3 evidence-based interventions to be tested in the cluster-randomized multifactorial pragmatic trial, Emergency Departments LEading Transformation of Alzheimer's and Dementia Care (ED-LEAD), designed to improve care for PLWD (see related editorial in this issue by Corita R. Grudzen).

### 1.1 | Preliminary Work: Primary Palliative Care for Emergency Medicine (PRIM-ER)

ECR draws from successful care management programs that consistently emphasize three elements: specific problem identification tools, psychosocial considerations essential for triadic encounters (PLWD, care partners, and clinicians), and structured post-discharge follow-up [16]. These elements guide the intervention's assessment approach and workflow design. Many of the assessment tools and justifications for their use are the direct culmination of these care management efforts in ambulatory settings [17].

ECR structures are additionally derived from the Primary Palliative Care for Emergency Medicine (PRIM-ER) framework [18]. PRIM-ER is a multicomponent primary palliative care intervention that has four key elements: (1) evidence-based multidisciplinary primary palliative care education, (2) simulation-based workshops on serious illness communication, (3) CDS, and (4) audit and feedback for ED clinical staff. We assessed the feasibility of the PRIM-ER intervention using a pragmatic, cluster randomized stepped-wedge design in 33 EDs across 18 health systems assigned to receive the intervention in a random sequential order. Adoption among emergency clinicians was high, with 2232 of 2715 eligible nurses (82%) and 879 of 1029 physicians and advanced practice providers (85%) completing all trainings [19].

## 2 | Aim

The overall goal of the ED-LEAD program is to conduct a pragmatic trial of re-engineered care for PLWD and their care partners (dyads) to improve post-discharge outcomes, as measured by decreased revisits and hospitalizations and increased days at home. The aim of this paper is to describe the conceptual basis and core components of the ECR intervention (one of 3 ED-LEAD programs) and the processes used to ensure implementation fidelity.

## 3 | Intervention Design

### 3.1 | Conceptual Model for the Intervention

**FIGURE 1 |:**
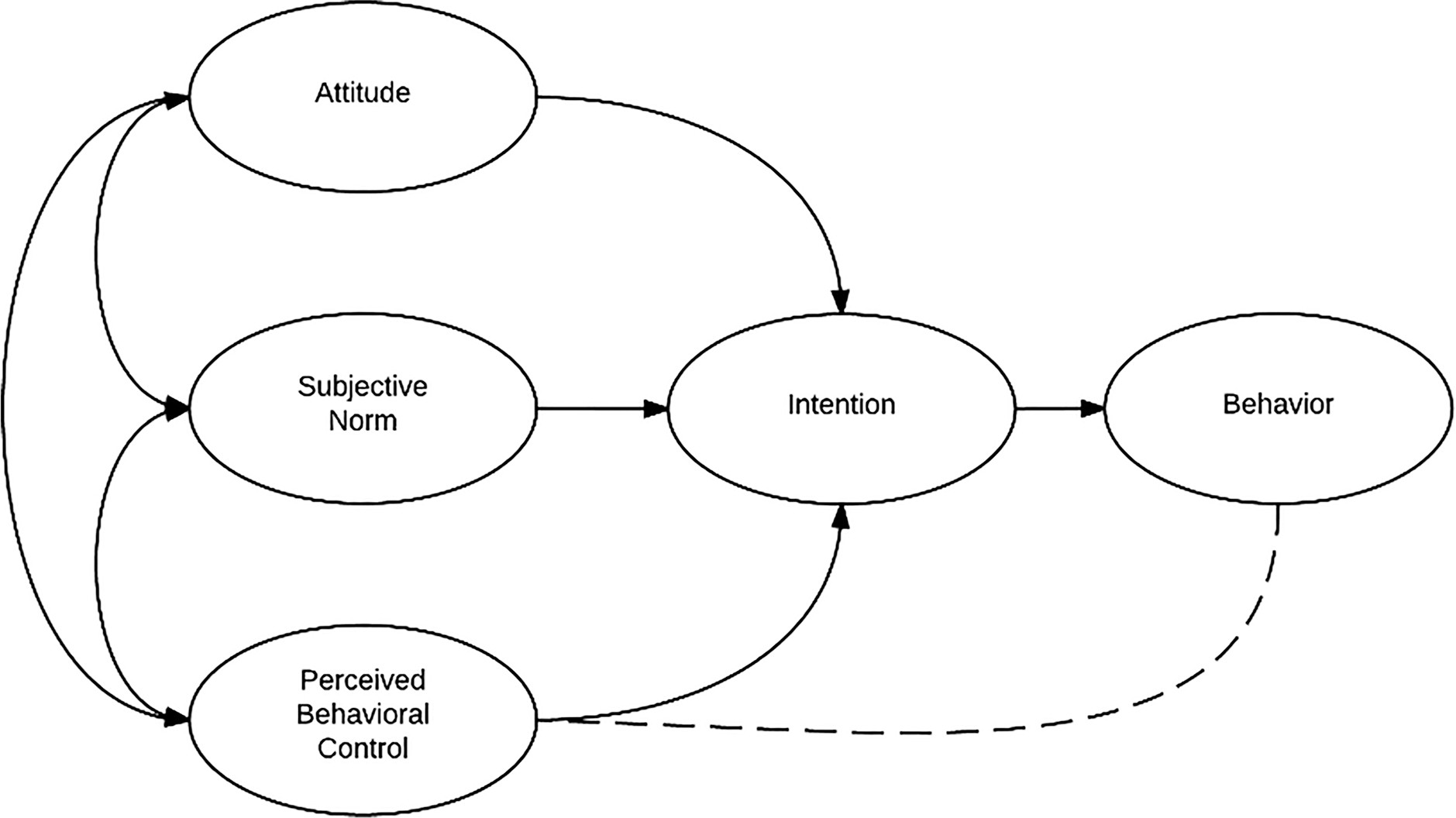
Theory of planned behavior.

The ECR program is grounded in a behavior change theory called the Theory of Planned Behavior, as described by Icek Ajzen (Figure 1) [20]. This theory, which has been applied to healthcare, specifies how an individual's intentions and behaviors are shaped by their attitude toward the behavior, subjective norms (perception about the behavior influenced by others), and perceived behavioral control (an individual's perceived ease or difficulty of performing the particular behavior). This theory was also shown to pertain to healthcare professionals' receptivity to and practice of palliative care, and a systematic review found that the Theory of Planned Behavior outperformed other social cognitive theories in predicting healthcare professionals' behavior [21]. This theory was also shown to pertain to palliative care [22] and thus has relevance for dementia care as a palliative care condition.

For ECR to succeed, providers' attitudes toward and comfort with dementia care must reflect a belief that improved provider–patient communication and simple interventions can positively impact the dyad's well-being. We address these attitudes both through training modules and by identifying and supporting discipline-specific clinical champions at each site who can model the ECR components and present dementia care success stories [23]. Altering subjective norms requires fostering a new ED scope of practice to discuss goals of care with dyads and establish community-based linkages. Demonstrating the importance of soliciting dyad-expressed care goals and tailoring recommendations and discharge plans to achieve those goals will encourage a new subjective norm for ED providers' scope of practice and the creation of effective discharge plans. Perceived behavioral control is based in self-efficacy theory, and in the dementia care context, is the comfort or ease with which emergency providers can discuss caregiving burdens, conduct a goals of care discussion, and address social services rather than confining discussion and attention to laboratory values or physical symptoms. Verbal scripts, simple talking points, and rationales for assessment strategies will help providers navigate these discussions more effectively.

### 3.2 | Description of the ECR Program

**FIGURE 2 |:**
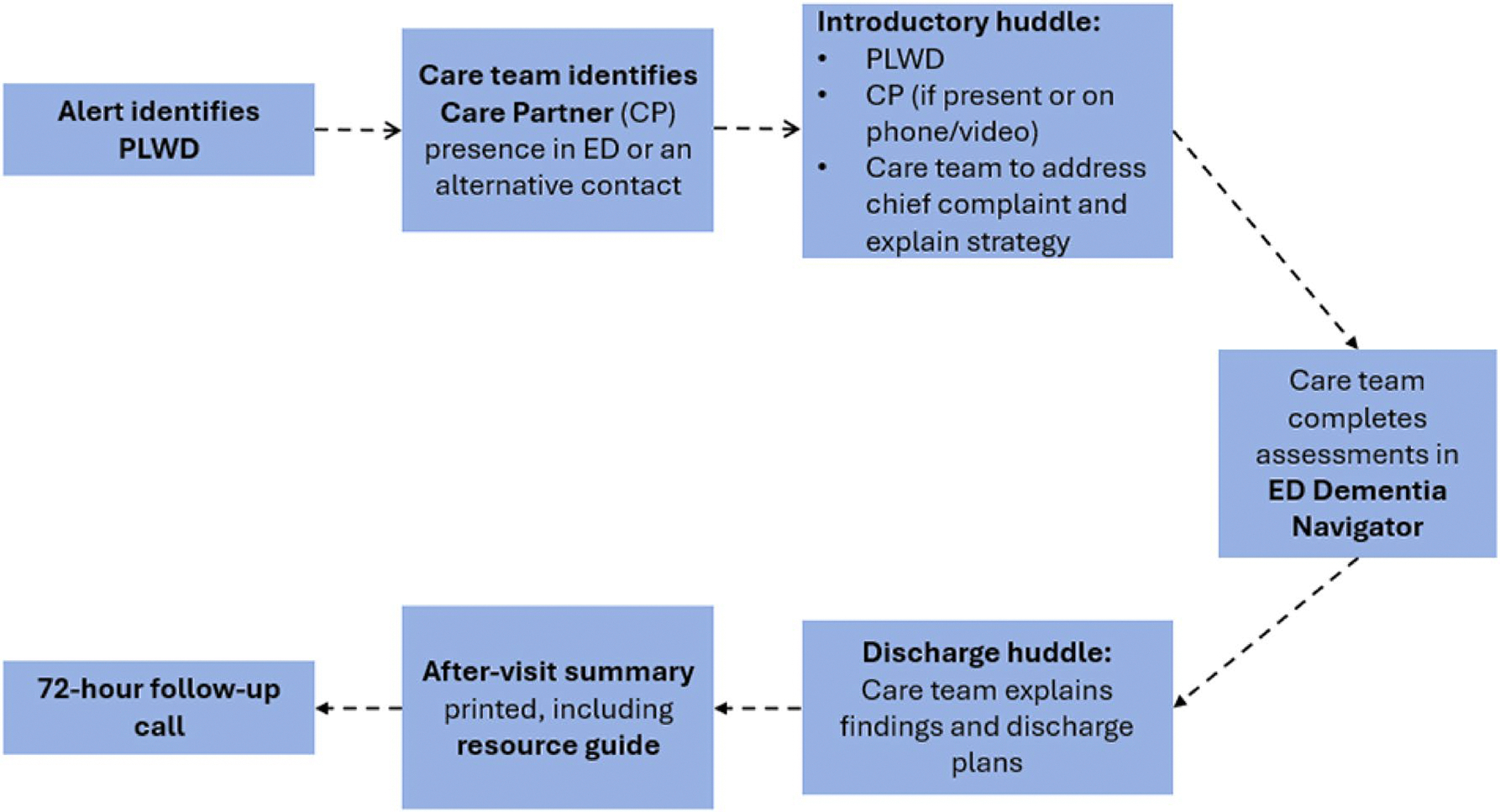
ECR workflow summary. **Bolded items** are supported by ECR-specific notes, prompts, or algorithms in the EHR. CP, care partner; ED, emergency department; PLWD, persons living with dementia.

ECR is comprised of multiple trial-tested dementia care management components [16, 24–26] embedded within an educational structure for emergency care providers. This includes CDS tools [23] for PLWD notifications, a shared structured worksheet or navigator with validated assessments, a locally curated resource guide for discharge referrals, and a 72 h post-visit phone call to reinforce community referrals. We importantly include nurses and social workers, in addition to emergency medicine physicians, as critical to in-ED assessments, developing effective discharge plans, and post-ED referrals. Attention to care partners, when available, is essential for effective dementia care, as they may be implementing the post-discharge recommendations and navigating daily care. CDS nudges and sample scripts are incorporated in the workflow to support ED team members in identifying and supporting such care partners. These procedures are bolstered by brief instructional videos deployed as asynchronous modules for required training before intervention launch and reminders for reinforcement. The overall workflow of the ECR program is shown in Figure 2, and each core ECR component is outlined below.

#### 3.2.1 | Training

To facilitate behavior change, ECR uses electronic learning (e-learning) [27] with both synchronous and asynchronous strategies, including scheduled and on-demand delivery of educational pearls to change attitudes toward dementia care, shift subjective norms, and reinforce the core components of the intervention. The full training curriculum is designed for all ED clinicians (physicians, advanced practice providers, nurses, and social workers) and takes ~1 h to complete. Emergency medicine residents are encouraged but not required to complete ECR training. The e-learning is based on principles of adult learning [28], reinforcing key concepts in dementia care, prompting attention through strategically placed questions, and using strategic repetition of concepts for better retention. The asynchronous series includes animated videos, interactive modules, and an EHR tutorial using site-specific EHR screenshots unique to each participating healthcare system. Continuing education units (1.5 CEU) are offered to participants who complete the training and post-test evaluation. Each ED is expected to train at least 75% (goal: 90%) of their ED staff (who work more than 50% time) by the ECR launch date.

ECR also provides synchronous virtual training, starting with town hall formats offered to multiple sites with a more general overview on all 3 ED-LEAD programs. There are also site-specific ECR meetings with site principal investigators (PIs), clinical champions, and program coordinators that include ECR-related presentations with time for questions and discussion. The same individuals are invited to monthly check-in meetings to review site-specific process reports, celebrate successes, and troubleshoot problems. These meetings offer the opportunity to reinforce dementia care and ECR content from the asynchronous training. The ECR team also provides 1-page tipsheets, huddle cards, and other handouts to be posted in strategic locations in each ED as training reminders.

#### 3.2.2 | Engaging Clinical Champions

A minimum of three clinical champions—physician, nurse, and social worker—are selected at each participating ED. Because ECR adds dyad-focused care processes to the standard ED workflow, successful implementation requires strong facility-level leadership to support the workforce and ensure procedural fidelity. Clinical champions lead their teams in this effort. Champions continuously assess feasibility and promote adoption of ECR processes within their team, educating their colleagues about the rationale and structure of the ED-LEAD study and the ECR intervention, specifically. They are a vital link for reevaluating resources and structuring on-site programming. Champions act as active clinical representatives for their team and relay site-specific feedback to project leadership.

#### 3.2.3 | Leveraging a National Dementia Care Management Support Service

CaringKind, a New York City–based nonprofit organization with over 40 years' experience connecting PLWD and care partners with information and resources, serves as the national care management support service for ECR to assist in the development of site-specific local resources addressing dyadic needs. Site-specific local resources are first identified and organized under specific categories of need and are uploaded into the EHR as a resource guide to be included in the PLWD's after-visit summary (AVS) (see Supporting Information S1 for a sample resource guide). CaringKind staff are also available to 72 h callers as well as site champions to field questions and support problem-solving. Additionally, they conduct scheduled monthly virtual meetings with 72 h callers to discuss challenging calls, successful interactions, and any local learning that can benefit others.

### 3.3 | Identification Strategy

Each ED uses EHR-incorporated analytics to flag any individual who has had an AD/ADRD ICD-10 diagnosis within the past 3 years in that health system (see Supporting Information S2 for a list of ICD-10 codes). These background analytics trigger an EHR alert for the treating team, indicating: (1) “This is a person living with dementia,” (2) “Identify the care partner,” and (3) “Please conduct a bedside huddle with other members of the care team.”

### 3.4 | CDS Tools

#### 3.4.1 | Implementation of ED Dementia Navigator

Depending on a site's EHR structure, the PLWD identification alert directs the ED care team to use the ED dementia navigator or flowsheet for information gathering. The navigator, available to any member of the care team, is a problem-based structured assessment tool in the EHR to help providers identify problems contributing to the ED visit or complicating discharge that might otherwise go undetected. Each navigator question collects information specific to the PLWD with structured prompts and the rationale for asking them. The navigator supports clinician efforts to efficiently identify actionable problems, improve in-ED care, and achieve a more effective discharge. Navigator sections include care partner information, goals of visit, memory and cognition, physical health and function, delirium screening, and risk appraisal. Better problem identification focused on high-frequency psychosocial issues may contextualize the presenting complaint and promote identification of resources to achieve a safer discharge. Site-specific EHR modifications facilitate easy access to the navigator in multiple ways. Depending on the needs and workflow of the given ED, this may include a link within the EHR alert, a link within the PLWD's chart, and a recognizable icon on the ED track board. See Table 1 for a list of assessments included in the ED dementia navigator.

#### 3.4.2 | Introductory Huddle

**FIGURE 3 |:**
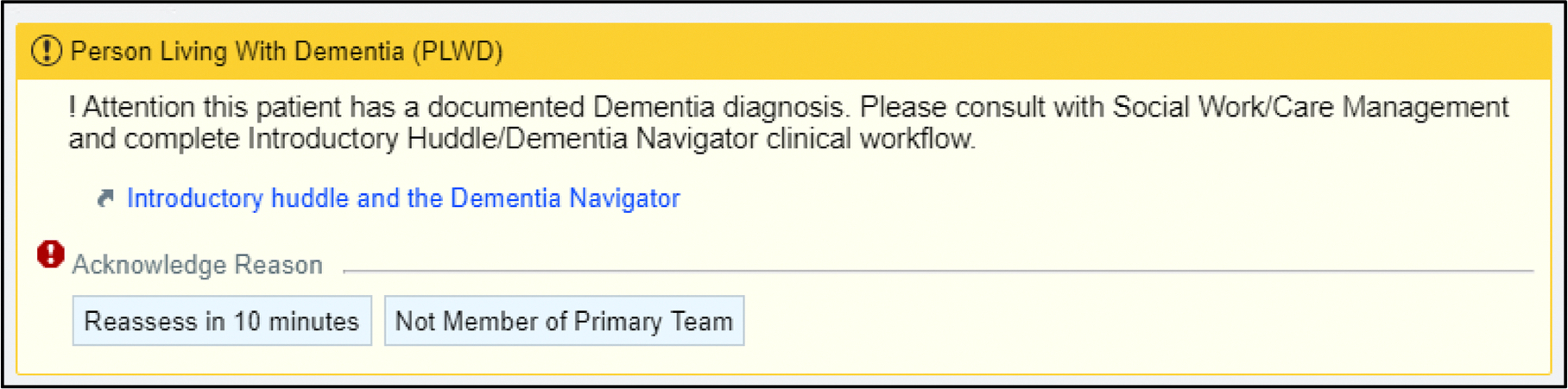
Sample introductory huddle alert.

An introductory huddle includes all members of the care team, the PLWD, and care partner when possible. Huddles can be in person or virtual (e.g., using EHR chat function). The introductory huddle should occur near the beginning of the ED visit. The goal is to review initial information gathered at admission, identify a care partner, increase trust in the care team, and use a collaborative process to solve problems. The huddle can also utilize the assessments within the navigator as an efficient data-gathering process. Responsibility for initiating and leading the huddle is a site-specific determination influenced by workflow and ED culture. The responsibility can be shared across disciplines, as all members of the care team opening the chart will receive the same alert that this is a PLWD and a huddle should be initiated. Once the huddle has taken place and has been documented, the initial EHR alert will disengage. Figure 3 shows a sample Introductory Huddle Alert.

#### 3.4.3 | Disposition Alert and Discharge Huddle

A disposition alert fires when a PLWD is being discharged home, prompting the team to hold a discharge huddle. This second huddle should include the dyad and members of the care team and should take place shortly before discharge. Care team members will be prompted by the Disposition Alert to conduct the huddle and document it in the ED Dementia navigator. The goal is to discuss the discharge plan, including resource guide recommendations and referrals to other programs. Each site-specific curated resource guide includes local resources, organized by problem, for ease of understanding and adherence. At the time of discharge, the PLWD receives their usual AVS plus the resource guide and any specific program referrals.

#### 3.4.4 | The 72-h Call: Relevant When an ED Is Randomized to ECR Only

When an ED is randomized to ECR only (no other post-discharge program), a brief, ≤ 30-min follow-up call takes place 72 h after the ED visit. The goal is to reinforce recommendations provided at ED discharge, ensure they are feasible for the dyad to implement, and address any concerns that were not discussed during the ED visit. These calls are structured by a 72 h call note template (Supporting Information S3), and callers (who may be nurses or social workers within that health system) receive the standard ED-LEAD training plus dedicated asynchronous training on the process and content of these calls. The 72 h call note is part of the medical record available to primary care providers and when direct communication would be helpful, the callers can reach out directly to primary care providers.

### 3.5 | Socialization and Marketing

New care processes and behavior change in busy clinical environments require on-site motivation, encouragement, and constant reminders. Promoting ECR as a new program to benefit both patients and providers is an essential strategy for effective implementation, which here requires both training and use of the CDS with fidelity. Our socialization plan includes email templates that site leadership teams (site PIs, champions, and coordinators) use to encourage and remind their staff to complete training and, post-launch, to support use of the navigator, huddles, and other ECR components. The study team provides each site with informational flyers and educational videos that can run as a loop in breakrooms. Finally, site leadership and champions select “swag” with the ED-LEAD logo (e.g., tote bags, mouse pads, badge reels, brain-shaped stress balls, and microfiber lens cloths) as both incentives for training completion and additional marketing tools.

### 3.6 | Intervention Monitoring

Throughout site implementation, intervention feasibility is monitored using a variety of data elements. We monitor staff training completion rates for each ED randomized to ECR, as training must be completed before the ED launch date. Within each site, once operational, we monitor ED disposition (numerator/denominator) for every PLWD who is flagged through the background EHR analytic algorithm. We track those who are discharged home, admitted, or transferred to another facility. For each flagged PLWD who is discharged home, we monitor the documented use of introductory and discharge huddles, care partner identification, navigator use, and completed 72 h post-discharge calls.

## 4 | Conclusion

We have described the conceptual basis for the ECR intervention within the ED-LEAD study, including training, participants, setting, intervention components, marketing and socialization, and the process and outcome measures monitored. Within the ECR intervention of this multifactorial pragmatic trial, many trial-tested as well as novel incorporated strategies address considerable gaps in the ED care of PLWD. These include timely identification of PLWD and their care partners, prompt recognition of key psychosocial issues contributing to or complicating presenting complaints, and matching identified problems with practical, community-based referrals post-discharge. Achieving uptake on use of recommended services after ED discharge has been challenging, and many barriers have been previously described [29]. Some novel strategies have increased uptake by bringing community service representatives into the ED, which might be difficult to implement on a national scale [30, 31]. ECR callers will be from that ED or health system, will have access to ED-visit EHR data, and are similar to current existing post-visit call strategies, which may increase uptake with more effective reinforcement of referral recommendations. By combining the strategies described above with innovative, practical training for ED providers aimed at changing attitudes, norms, and behaviors, we may improve discharge processes and outcomes for PLWD and their care partners.

## Supplementary Material

Supplement

Additional supporting information can be found online in the Supporting Information section. **Supporting Information: S1**. Sample resource guide. **Supporting Information: S2** ICD-10 Codes to Identify AD/ADRD. **Supporting Information: S3** 72 h call note template.

## Figures and Tables

**TABLE 1 |:** Elements of dementia navigator built into the EHR.

Element	Content	Justification

Introductory huddle^a,b^	How did the huddle take place?	A huddle ensures shared understanding.
Care partner^a,b^	Does the patient have an identified care partner?	Identifying a care partner eases transition home.
Goals of visit	“What is most important to you for your care?”	Knowing care goals can help match resources at discharge.
Memory and cognition	“Have you experienced any challenges with thinking or memory?”	Diagnosis may not have been previously discussed.
Physical health and function	Is the patient having trouble with vision, hearing, bathing, toileting, dressing, walking, eating, and/or managing personal hygiene?	Identifying hearing, vision, or other functional challenges offers opportunities for effective discharge and can reduce risks.
Delirium screening (FAM-CAM)	“During this week, have you noticed any changes in his/her thinking or concentration, such as being less attentive, appearing confused or disoriented?”	Delirium is common and is a red flag for possible serious illness not otherwise detected.
Risk appraisal	Does the patient have access to dangerous objects?	Identifying safety factors can lead to a safer discharge with mobilizing social work.
Discharge huddle^a^	How did the huddle take place?	Huddle creates opportunity for providing most appropriate and feasible recommendations.

Abbreviations: EHR, electronic health record; FAM-CAM, family confusion assessment method.

a Some CDS builds in the EHR incorporate these elements within their navigator, whereas other builds have these as separate elements.

b The introductory huddle and care partner components of the CDS navigator are strongly recommended as a hard stop or visual cue.

## Summary

- Key points
  - Emergency departments (EDs) are not well equipped to manage presenting problems of persons living with dementia (PLWD).
  - Focused training, clinical decision supports, and structured follow-up calls 72 h after discharge can encourage new workflows and improve quality of care for PLWD.
  - Close monitoring for audit and feedback ensures continuous quality improvement and may increase fidelity to the processes that will be implemented in Emergency Care Redesign.
- Why does this paper matter?
  - New strategies to improve care for and outcomes of PLWD who visit EDs are urgently needed.
  - This paper provides a blueprint for an ED strategy that may improve the lives of millions of PLWD and their family care partners.
