# Supplementary material for: Emergency Departments Leading the Transformation of Alzheimer's and Dementia Care: Emergency Care Redesign
Source: J Am Geriatr Soc. 2026 Mar 25;74(7):1864–71. doi: 10.1111/jgs.70394 (PMC13314118; doi:10.1111/jgs.70394)
Supplement: Supplementary file 1 — Supporting Information: S1. Sample resource guide. Supporting Information: S2 ICD‐10 Codes to Identify AD/ADRD. Supporting Information: S3 72 h call note template. [file JGS-74-1864-s001.pdf]

Supplemental Material S1. Sample resource guide
Supplemental Material S2. ICD-10 Codes to Identify AD/ADRD
Supplemental Material S3. 72-hour call note template

Supplemental Material 1

Community Resources for Older Adults

Below is a list of local and national organizations that provide help and resources for older adults.

Do you need help with...?	To connect with services for…	Reach out to these local resources:
**Physical Health**	Primary care, geriatricians, neurologists	• ***Site-specific listings***<br>• **Call Primary Care Doctor**<br>• **Contact your health insurance**
**Mental Health**	Mental Health Providers including psychiatrists (doctors) and other therapists / counselors	• ***Site-specific listings***<br>• **Contact your health insurance**<br>• **Crisis Line**<br>○ Call or Text 988<br>• **211 (for information on community clinics)**
**Dentistry**	Dentists (oral care)	• ***Site-specific listings***<br>• **Contact your health insurance**<br>• **Local Area Agency on Aging**
**Vision**	Eye Doctors, visual aids, and other services	• ***Site-specific listings***<br>• **Contact your health insurance**<br>• **Local Area Agency on Aging**
**Hearing**	Hearing Specialists (audiologists), hearing aids	• ***Site-specific listings***<br>• **Contact your health insurance**<br>• **Local Area Agency on Aging**
**Transportation**	Public transportation and driving	• ***Site-specific listings***<br>• **Contact your health insurance**<br>• **Local Area Agency on Aging**
**Mobility**	Assistive Devices (wheelchairs, canes, and walkers)	• ***Site-specific listings***<br>• **Contact your health insurance**<br>• **Local Area Agency on Aging**<br>• **Alzheimer's Association**<br>○ Phone number: 1-800-272-3900<br>○ Website: www.alz.org
**Home Care**	Home safety assessment, home care services	• ***Site-specific listings***<br>• **Local Area Agency on Aging**<br>• **Alzheimer's Association**<br>○ Phone number: 1-800-272-3900<br>○ Website: www.alz.org

**Long-Term Care**<br>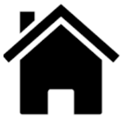	Adult/Assisted Living Facilities, Skilled Nursing Facilities	• ***Site-specific listings***<br>• **211 (for information on facilities)**<br>• **Local Area Agency on Aging**
**Food Assistance**<br>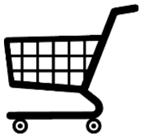	Meal delivery, groceries, nutrition	• ***Site-specific listings***<br>• **Supplemental Nutrition Assistance Program (SNAP):**<br>○ Phone number: 1-800-792-9773
**Benefits**<br>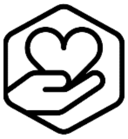	Medicare, Medicaid, Social Security, Veteran Affairs	• ***Site-specific listings***<br>• **Medicare Rights Center:**<br>○ Phone number: 1-800-333-4114<br>○ Website: https://www.medicarerights.org/<br>• **Medicaid Helpline:**<br>○ Phone number: 1-800-541-2831<br>• **Social Security Administration:**<br>○ Phone number: 1-800-772-1213<br>○ Website: www.ssa.gov<br>• **Veteran Affairs**<br>○ Phone number: 1-800-772-1213<br>○ Website: https://www.va.gov/
**Legal**<br>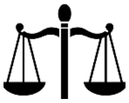	Adult Protective Services, elder abuse, elder law, identity theft	• ***Site-specific listings***<br>• **Adult Protective Services**<br>○ Phone number: 1-800-898-4910
**Social Engagement**<br>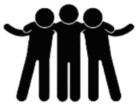	Support groups, language assistance, senior centers	• ***Site-specific listings***<br>• **CaringKind:**<br>○ Phone number: 646-744-2900<br>○ Website: www.caringkindnyc.org
**Comfort Care**<br>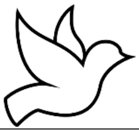	Palliative care, hospice care	• ***Site-specific listings***<br>• **Contact your health insurance**<br>• **Local Area Agency on Aging**
**Care Partner Support**<br>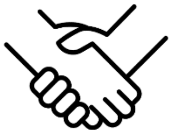	Respite care, support groups	• ***Site-specific listings***<br>• **CaringKind:**<br>○ Phone number: 646-744-2900<br>○ Website: www.caringkindnyc.org<br>• **Alzheimer's Association**<br>○ Phone number: 1-800-272-3900<br>○ Website: www.alz.org

Other National and Local Organizations

**National Institute on Aging (NIA)**<br>• The NIA leads the federal government in research on aging and Alzheimer’s disease and related dementias. You can order and download free publications about healthy aging and age-related diseases and conditions.<br>• Website: https://order.nia.nih.gov/<br>• Phone: 800-222-2225
**Geriatric Care Managers**<br>• A geriatric care manager works with an individual and / or their family by assessing the current situation, identifying needs, and creating a care plan. They will also connect you to services needed. GCMs usually charge a general assessment fee and then charge by the hour. Note: They can be expensive, and insurance may not cover the cost of their services.<br>• Website: https://www.aginglifecare.org/
**National Academy of Elder Law Attorneys (NAELA)**<br>• This is a professional association of attorneys dedicated to improving the quality of legal services provided to older adults and people with disabilities. Dementia organizations may have recommended lists of Elder Law Attorneys.<br>• Website: https://www.naela.org/
**Area Agency on Aging**<br>• These agencies address the needs and concerns of all older persons at the regional and local levels.

Supplemental Material 2

Supplemental Material S2. ICD-10 Codes to Identify AD/ADRD

**Cohort**	**ICD-10 Codes**
Alzheimer's Disease and Alzheimer's Disease-Related Dementias	F01.50, F01.51, F02.80, F02.81, F03.90, F03.91, F04, G13.8, F05, F06.1, F06.8, G30.0, G30.1, G30.8, G30.9, G31.1, G31.2, G31.01, G31.09, G94, R41.81, R54

Source: McCarthy et al. 2021

AD/ADRD = Alzheimer's Disease and Alzheimer's Disease-Related Dementias

Supplemental Material 3

Supplemental Material S3. 72-Hour Call Template

This note template can be accessed via a dot/SmartPhrase and should be completed during the 72-hour call.

For Care Partner of PLWD

- Care partner present on call **(dropdown menu)**
  - Yes
  - No
- Care partner living situation **(dropdown menu)**
  - CP lives with PLWD
  - CP does not live with PLWD
- Relationship **(dropdown menu)**
  - Spouse/partner/significant other
  - Adult child
  - Sibling
  - Other family member
  - Neighbor
  - Friend
  - Other **(open answer)**
- Same care partner present during ED visit **(dropdown menu)**
  - Yes
  - No

Review goals of care and recommendations

- Medication changes:
  - {DOES/DOES NOT} have refills for all
  - {DOES/DOES NOT} feel able to afford monthly medication costs
  - STOPPED *** because it was causing ***
  - CHANGED *** because it was causing ***; instead, take it as follows: ***
  - STARTED the following new medication to treat *** and take it as follows: ***
- Referrals already contacted **(dropdown menu)**
  - YES, PLWD contacted already
  - NO, PLWD did not contact
- We suggest you contact the following resources for continued care **(select all that apply)**
  - **ADD SITE-SPECIFIC RECOMMENDATIONS**
- Call status **(dropdown menu)**
  - Call attempted and left voicemail (note pending)
  - Call completed
  - PLWD ineligible (nursing home, etc.)
  - PLWD unreachable
